# Mechanistic Insights Into Metal Binding in the Aggregation Pathway of an Immunoglobulin Light Chain Protein

**DOI:** 10.1002/cbic.70460

**Published:** 2026-07-14

**Authors:** Tejaswini Pradhan, Riddhiman Sarkar, Joshua Leonhard‐Preu, Adrian F. Schnell, Nadine Schwierz, Bernd Reif

**Affiliations:** ^1^ Bayerisches NMR Zentrum (BNMRZ) at Department of Bioscience TUM School of Natural Sciences Technische Universität München Garching Germany; ^2^ Helmholtz‐Zentrum München (HMGU), Deutsches Forschungszentrum für Gesundheit und Umwelt, Institute of Structural Biology (STB) Neuherberg Germany; ^3^ Centre of Biomedical Research (CBMR) Sanjay Gandhi Post Graduate Institute of Medical Sciences (SGPGIMS) Campus Lucknow Uttar Pradesh India; ^4^ Academy of Scientific and Innovative Research (AcSIR) Ghaziabad Uttar Pradesh India; ^5^ Institute of Physics Computational Biology University of Augsburg Augsburg Germany

## Abstract

Cardiac antibody light chain (AL) amyloidosis is characterized by the deposition of light chain (LC) variable domain (V_L_) protein in the heart. Transition metals such as copper and zinc have been suggested to enhance the toxicity of circulating soluble LCs. We show here how divalent metal ions influence the aggregation behavior of the patient‐derived λ‐III AL variable domain FOR005. We characterize metal binding across dimeric, monomeric, oligomeric, and fibril aggregation states, using solution‐ and MAS solid‐state NMR spectroscopy, as well as molecular dynamics (MD) simulations. Zn^2+^ and Cu^2+^ reduce the lag time for fibril formation. In the dimer, the Cu^2+^ binding site involves the side chains of residues D91 from protomer 1 and H96 from protomer 2. The accelerated aggregation kinetics induced by Cu^2+^ arises from differential stabilization of oligomeric intermediate states, either through depletion of off‐pathway intermediates or shortened lifetimes of on‐pathway species. We find that Cu^2+^ does not bind to a specific site but interacts nonspecifically with the protein fibrils. We hypothesize that a decrease of the cytosolic free pool of ions perturbs the cellular ion homeostasis in cardiomyocytes which in turn can result in dysregulated metabolic function.

## 1 Introduction

Protein misfolding and aggregation are causative for a broad class of human diseases, including the systemic disorder immunoglobulin light chain (AL) amyloidosis. In this disease, monoclonal plasma cells secrete misfolding‐prone light chain (LC) antibody proteins that circulate in serum, form soluble oligomers, and deposit as insoluble amyloid fibrils in target organs. Insoluble, fibrillar deposits are the major culprit in cardiac AL amyloidosis. However, also, soluble LC species are directly cytotoxic to cardiomyocytes and contribute to progressive diastolic dysfunctions [1, 2]. Despite substantial progress, the structural basis of LC misfolding and the mechanisms by which soluble intermediates cause toxicity remain poorly understood.

The misfolding pathway of LC variable domains (V_L_) is thought to proceed through a series of discrete intermediates [3]. In solution, the protein predominantly exists as a noncovalent dimer. Monomerization is an early step in the misfolding process, followed by partial unfolding and formation of soluble oligomeric species whose structures remain poorly defined. These oligomers subsequently convert into β‐sheet–rich amyloid fibrils. Understanding the energetics and structural features of each step is critical for elucidating how sequence, environment, and cofactors modulate aggregation.

Among the extrinsic factors influencing amyloid formation, divalent metal ions have emerged as important modulators of protein misfolding. Transition metals such as copper (Cu^2+^) and zinc (Zn^2+^) are essential for numerous cellular processes, including enzymatic catalysis, redox balance, and signaling. Dysregulation of their homeostasis is implicated in several protein aggregation disorders, including Alzheimer’s, Parkinson’s, and prion diseases, where metals can accelerate aggregation or alter fibril morphology [4, 5]. Cu^2+^ and Zn^2+^ ions can coordinate to histidine, aspartate, and glutamate residues, stabilizing transient conformations or perturbing native interactions that maintain protein stability. Similar mechanisms may operate in immunoglobulin LCs, which show sequence‐dependent metal binding often involving the CDRs and N‐terminal regions.

In the context of AL amyloidosis, circulating LC proteins can bind divalent cations and thereby potentially perturb cellular ion homeostasis [6, 8]. Studies have shown that exposure of cardiomyocytes or cardiac fibroblasts to amyloidogenic LC proteins induces oxidative stress, mitochondrial dysfunction, and apoptosis [9, 10]. Reactive oxygen species (ROS) formation in these systems is metal‐dependent, suggesting that metal–LC interactions may directly contribute to cytotoxicity.

Given the central role of Cu^2+^ and Zn^2+^ in cellular physiology and their ability to coordinate to side chains commonly present in antibody variable domains, it is plausible that these ions modulate the LC misfolding pathway. However, the molecular mechanisms by which metal ions affect the conformational equilibria and aggregation kinetics of LC proteins remain unclear. Specifically, it is not known which intermediate states—native dimers, partially unfolded monomers, soluble oligomers, or fibrils—are most relevant for metal binding, nor how metal coordination alters the interconversion between these species.

To understand the effect of Cu^2+^ and Zn^2+^ on LC misfolding, it is essential to dissect their interactions along the entire aggregation pathway. Metal ions may either destabilize the native dimeric state, thereby promoting monomerization and misfolding, or stabilize partially folded intermediates and fibrils through specific coordination sites. These interactions could shift the population of on‐pathway and off‐pathway species, ultimately affecting aggregation kinetics and fibril stability. In this study, we investigate the interactions of Cu^2+^ and Zn^2+^ with the patient‐derived λ‐III AL variable domain FOR005 and its corresponding germline (GL) protein.

Using solution‐ and solid‐state nuclear magnetic resonance (NMR) spectroscopy and molecular dynamics (MD) simulations, we characterize metal binding to distinct conformational states of the LC protein, including monomers, dimers, oligomers, and fibrils.

## 2 Materials and Methods

### 2.1 Protein Expression and Purification

Recombinant protein productions were purified as described previously [11, 12]. Briefly, E. coli BL21 with a pET28(b+) vector encoding the FOR005 gene were cultured in LB medium for biophysical experiments and in minimal medium for NMR experiments. Bacterial expression was induced with 1 mM IPTG at OD 0.6–0.8 followed by overnight expression at 37°C. Cells were harvested, and inclusion bodies were isolated. Solubilized protein from inclusion bodies was subjected to anion exchange chromatography followed by refolding using redox agents and a 3.5 kDa dialysis bag. Finally, pure protein was obtained using size exclusion chromatography. Molecular mass was confirmed by using SDS‐PAGE and ESI‐MS. To produce isotopically labeled protein, ^15^NH_4_Cl and ^13^C‐glucose were employed as nitrogen and carbon sources, respectively. All mutants were purified in the same way as the patient protein. In all biophysical and NMR experiments, the protein was solubilized in a 20 mM phosphate buffer [NaH_2_PO_4_ + Na_2_HPO_4_] containing 50 mM NaCl. The pH was adjusted to 6.5.

### 2.2 Thioflavin T (ThT) Experiments

Fibril formation kinetics was measured using a ThT assay [13]. Prior to the ThT assay, protein solutions were centrifuged for 3–4 h at 45,000 rpm to remove preformed aggregates using a table‐top ultra‐centrifuge (Beckman Optima MAX‐E equipped with a TLA‐45 fixed angle rotor). 0.05% sodium azide was added in all samples to avoid bacterial growth. Experiments were carried out using a fluorescence spectrometer (FLUOstar Omega, BMG Labtech), employing a fluorescence excitation and emission wavelength of 448 and 482 nm, respectively. The FLUOstar is equipped with 96‐well plates (Thermo Fisher Scientific) accommodating 150 μL in each well. All protein samples were incubated with 25 μM ThT with constant agitation using double orbital shaking (300 rpm). Divalent metal ion (copper chloride, zinc chloride (II)) stocks were prepared in water, and the equivalents added in this experiment are mentioned in the figure and/or in the main text. In all experiments, the sample temperature was kept constant at 37°C. The plates were sealed with polyester sealing film (Starlab) to avoid evaporation. For each protein and concentration, three replicates have been recorded.

### 2.3 Transmission Electron Microscopy (TEM)

TEM experiments were performed in order to visualize fibrils. Fibrils were washed with distilled water to remove phosphate buffer prior to grid formation. Formvar/carbon 300‐mesh copper‐coated carbon grids (Electron Microscopy Sciences) were exposed first for 30 s to an argon atmosphere. Five microliters of fibril solution was then added to the grids and incubated for 1 min. Grids were subsequently washed with water and dried using filter paper. For staining, 5 μL of uranyl acetate (2%) was added for up to 30 s. Extra stain was removed from the grid using filter paper. Grids were visualized in TEM employing a JEOL 1400 plus microscope.

### 2.4 Fibril Sample Preparation for Solid‐State NMR

Fibril samples were prepared as described previously [12, 14]. Seeded FOR005 fibrils were prepared using 50 μM protein and 5% ex vivo seeds that were incubated for 2 weeks. To prepare the copper containing fibril sample, a tenfold molar excess of CuCl_2_ was added to preformed seeded FOR005 fibrils. Protein aggregates were first centrifuged to reduce the volume to approx. 500 mL. Subsequently, the fibril slurry was sedimented for 1 h into a 1.9‐mm thin‐wall ZrO_2_ MAS rotor (Bruker Biospin), using a spiNpack 1.9‐mm rotor filling tool (Giotto Biotech) and a L‐100 XP ultracentrifuge (Beckman Coulter) equipped with a SW 32 Ti swinging bucket rotor operating at 28,000 rpm at 4°C.

### 2.5 Protease Digestion of Fibrils

Fibrils were subjected to proteinase K digestion to assess their proteolytic stability [15]. Prior to digestion, fibrils were washed with milli‐Q water. Twenty‐two microliters of 10 × TCB buffer (200 mM Tris, 1.4 M NaCl, 20 mM CaCl_2_, 1% w/v NaN_3_, pH 8.0) was added to achieve a final reaction volume of 220 μL with a fibril protein concentration of 200 μg/mL. The reaction mixture was incubated at 37°C in a thermoblock, and digestion was initiated by the addition of 0.4 μL proteinase K (Roche). The proteinase K assay was performed using 0.692 μM proteinase K and 8.6 μM FOR005, at a molar ratio of proteinase K to FOR005 of 1:12.5 [15]. An initial aliquot was collected immediately prior to addition of proteinase K. Subsequent aliquots were taken at 5, 10, 30, and 60 min after addition of the enzyme. Each aliquot was immediately mixed with 2 μL of protease inhibitor solution (200 mM phenylmethylsulfonyl fluoride (PMSF) in methanol; Carl Roth), incubated at room temperature for 10 min, and then snap‐frozen in liquid nitrogen. After all time points were collected, samples were thawed at room temperature and analyzed by SDS‐PAGE using 4%–12% Bis‐Tris NuPAGE gels with MES SDS running buffer (Invitrogen).

### 2.6 Solution‐State NMR Experiments

All NMR experiments were conducted at 25°C, employing 1200 MHz and 800 MHz Bruker Avance III spectrometer equipped with cryogenic triple resonance probes. Assignments of the NMR backbone chemical shifts were transferred from previously assigned spectra [3]. NMR data was acquired and processed using Topspin 4.1.1 (Bruker) software, and all spectral analyses were performed using CcpNMR‐V2 software [16].

To probe the effect of divalent metal ions, NMR spectra were recorded using a 50 μM protein solution supplemented with metal ion equivalents as specified in the main text. For the time‐dependent NMR aggregation assay, two samples were prepared: first, a 50 μM FOR005 reference protein solution, and second, a 50 μM protein solution supplemented with a fourfold molar excess of CuCl_2_. ^1^H,^15^N HSQC spectra were recorded on a daily basis for 7 days for both samples. To prevent bacterial contamination during aggregation, 0.02% sodium azide was added to the protein samples on day 1. Chemical shift changes in ^1^H,^15^N HSQC spectra were calculated using the formula

$$\text{Δ}\delta_{\mathrm{NH}}=\sqrt{{\text{(}\text{Δ}\delta^{1H})}^{2}+\frac{1}{25}{\text{(}\text{Δ}\delta^{15N})}^{2}}$$

### 2.7 Solid‐State NMR Experiments

All solid‐state NMR experiments were carried out at an external magnetic field of 17.6 T (corresponding to a ^1^H Larmor frequency of 750 MHz). 2D ^13^C,^13^C correlation experiments were acquired using DARR mixing using a mixing time of 50 ms [17]. For 2D ^13^C,^15^N correlation experiments, conventional NCA experiments were recorded using a mixing time of 50 ms [18]. The fibril NMR chemical shift assignments were obtained previously [14]. The MAS rotation frequency was adjusted to 16.6 kHz, and the sample temperature was maintained at 10°C using a cooling gas flow rate of 550 L/h. Samples with and without CuCl_2_ were measured in the same spectrometer using similar parameters. CcpNMR‐V2 was employed for the analysis of all solid‐state NMR experiments [19].

### 2.8 Simulation Setup and Parameters

To analyze the effect of Cu^2+^ ions on the FOR005 dimer (PDB:5L6Q), we prepared four different simulation setups. In the control setup, we used 50 mM NaCl without Cu^2+^ ions. In the other three setups, we used 50 mM NaCl and placed Cu^2+^ ions either at binding site (BS) I, II, or at both sites. The initial placement of the Cu^2+^ ions was based on the BS of the Zn^2+^ atoms in 5L6Q. During energy minimization, position restraints were applied on the Cu^2+^ ions. Additionally, we modeled the structure λ‐III LC amyloid fibril (PDB: 6Z10), using LocalColabFold [20, 21] to reconstruct missing residues.

For each dimer setup, we performed three independent runs at 300 K and three runs at 400 K. We performed six additional 200‐ns simulations at 300 K for the dimer and three for the fibril, each solvated in 150 mM CuCl_2_, without placing ions in BS I or II, to test whether ions spontaneously occupy the experimental BS within the MD timescale. In addition, we performed simulations of the FOR005 monomer in 150 mM CuCl_2_ and 50 mM NaCl.

To predict protonation states of titratable residues, the structures were preprocessed using the H++ webserver (http://newbiophysics.cs.vt.edu/H++), assuming a salinity of 50 mM, a pH of 6.5, an internal dielectric constant of 10, and an external dielectric constant of 80. Afterward, the protein structure was placed in the center of a tetragonal box with a minimum distance of 2.0 nm between the protein and the boundaries of the simulation box. The systems were solvated with OPC water and either 50 mM NaCl or 150 mM CuCl_2_. The dimer systems contained roughly 73,000 particles in a box of (7.7 × 10.5 × 8.2) nm^3^, and the fibril systems consisted of approximately 169,000 particles with box dimensions of (13.7 × 12.1 × 9.1) nm^3^. For an optimal combination with OPC water, we used Mamatkulov–Schwierz ion parameters [22] for Na^+^ and Loche–Bonthuis parameters [23] for Cl^−^ ions. To describe Cu^2+^ ions, we used the Li–Merz [24] parameters. Protein force field parameters were taken from the AMBER ff19SB force field [25], and protein topology files were generated using AmberTools24 [26].

MD simulations were performed using GROMACS 2024.5 [27]. The initial energy minimization of the system was carried out using a steepest descent algorithm, followed by a 1 ns NVT equilibration using the Berendsen thermostat [28] and 1 ns NPT equilibration employing the Berendsen barostat [29] as well as the velocity‐rescaling algorithm [30]. Finally, the production runs of the systems were carried out using the leap‐frog algorithm with a 2 fs integration time step in the NPT ensemble where isotropic pressure coupling was handled by the Parrinello–Rahman barostat [31] with a reference pressure of 1 bar and a coupling constant of 5.0 ps, while temperature coupling was realized through the velocity‐rescaling algorithm with a reference temperature of either 300 or 400 K. We applied periodic boundary conditions to the system in all directions. Long‐range interactions were handled by the Particle Mesh Ewald method with a 1.2 nm cutoff, while van der Waals interactions were handled with a cutoff value of 1.2 nm. We used a Fourier spacing of 0.12 nm and applied long‐range dispersion corrections for energy and pressure. All hydrogen bonds were constrained with the LINCS algorithm [28].

### 2.9 Snapshots

Snapshots of the dimer and fibril systems were created using PyMOL 3.1.3 (The PyMOL Molecular Graphics System, Version 3.0 Schrödinger, LLC).

### 2.10 Contact Frequencies

Contact frequencies for the Cu^2+^‐dimer complex and the fibril CuCl_2_ systems were calculated using MDAnalysis 2.9.0 [32] and the Contact Map Explorer Python package (https://github.com/dwhswenson/contact_map) which is built upon the MDTraj package [33].

### 2.11 MMPBSA

MMPBSA calculations on the FOR005 dimer were done using MMPBSA.py 14.0 [34] as a part of AmberTools 24. For the calculations, the representative structures of the main cluster for each system (Cu^2+^ in BS I, II, and I+II) were taken as initial structures for additional MD simulations. These structures were solvated in 150 mM NaCl solution, the systems were neutralized with Na^+^ and Cl^−^ counter ions and then underwent a three‐stage energy minimization until convergence was reached using the steepest descent algorithm with a decreasing step size for each of the three stages. In the MMPBSA calculations, entropic contributions arising from conformational changes upon binding are typically neglected. To ensure consistency with this approximation, position restraints were applied in the MD simulations, using representative structures obtained from clustering with ions placed in their respective BS. Specifically, to preserve the BS geometry, position restraints of 10,000 kJ mol^−1^ nm^−2^ were imposed on protein heavy atoms and Cu^2+^ ions during energy minimization. Subsequently, the systems underwent 1 ns of NVT using the Berndsen thermostat with a coupling constant of 0.1 ps. This was followed by 1 ns of NPT equilibration using the Berendsen barostat, with a pressure coupling constant of 2 ps and a reference pressure of 1 bar, as well as the velocity‐rescaling thermostat with a temperature coupling constant of 0.1 ps. During NVT and NPT equilibration and the 5 ns production run, position restraints of 1000 kJ mol^−1^ nm^−2^ were applied to protein heavy atoms and Cu^2+^ ions. For the production run, isotropic Parrinello–Rahman pressure coupling with a coupling constant of 5 ps was combined with the velocity‐rescaling algorithm. For the conversion of GROMACS files, the AMBER programs parmed and cpptraj were used. Two hundred fifty frames, stripped from water and Na^+^ and Cl^−^ ions, were taken from the 5 ns trajectories. The FOR005 dimer was specified as the receptor and Cu^2+^ ions as ligands, allowing for the calculation of binding free energies in the absence and presence of Cu^2+^ ions from a single trajectory. The ionic strength was matched to the MD simulations at 150 mM. Reported means and standard deviations were taken directly from MMPBSA.py 14.0. The reported relative binding energy changes $\text{Δ}E_{\mathrm{rel}}$ were normalized to the Cu^2+^‐bound state as follows:

$$\text{Δ}E_{\mathrm{rel}}=1-\frac{\;E_{\mathrm{bind}}^{\;wo\;Cu^{2+}}}{E_{\mathrm{bind}}^{\;w\;Cu^{2+}}}$$

### 2.12 RMSF, RMSD, and Dihedral Angles

RMSF and RMSD values were calculated with GROMACS after backbone alignment and in reference to 5L6Q. Calculations of the dihedral angles Φ and Ψ were done using MDAnalysis 2.8.0 [32, 35, 36] where indicated fully allowed regions are based on the calculations by Ramachandran, Ramakrishnan, and Sasisekharan [37].

## 3 Results

FOR005 is a patient‐derived λ‐III LC sequence [12, 38]. The respective GL is mutated at five positions, namely at residues S31Y, F48Y, R49G, S51N, and A94G (mutations indicate transitions from patient to the GL protein). The importance of the individual mutants with respect to aggregation propensity and thermodynamic stability has been studied recently [39]. It is the aim of this work to characterize the interactions of divalent metal ions with dimeric, monomeric, oligomeric, and fibrillar LC variable domain FOR005. To pursue this question, we first analyzed the influence of metal ions on the aggregation behavior and subsequently characterized the interactions of the ions using solution‐state and MAS solid‐state NMR and MD simulations.

### 3.1 Aggregation Kinetics of FOR005 With Cu^2+^ and Zn^2+^ by ThT Assays

**FIGURE 1:**
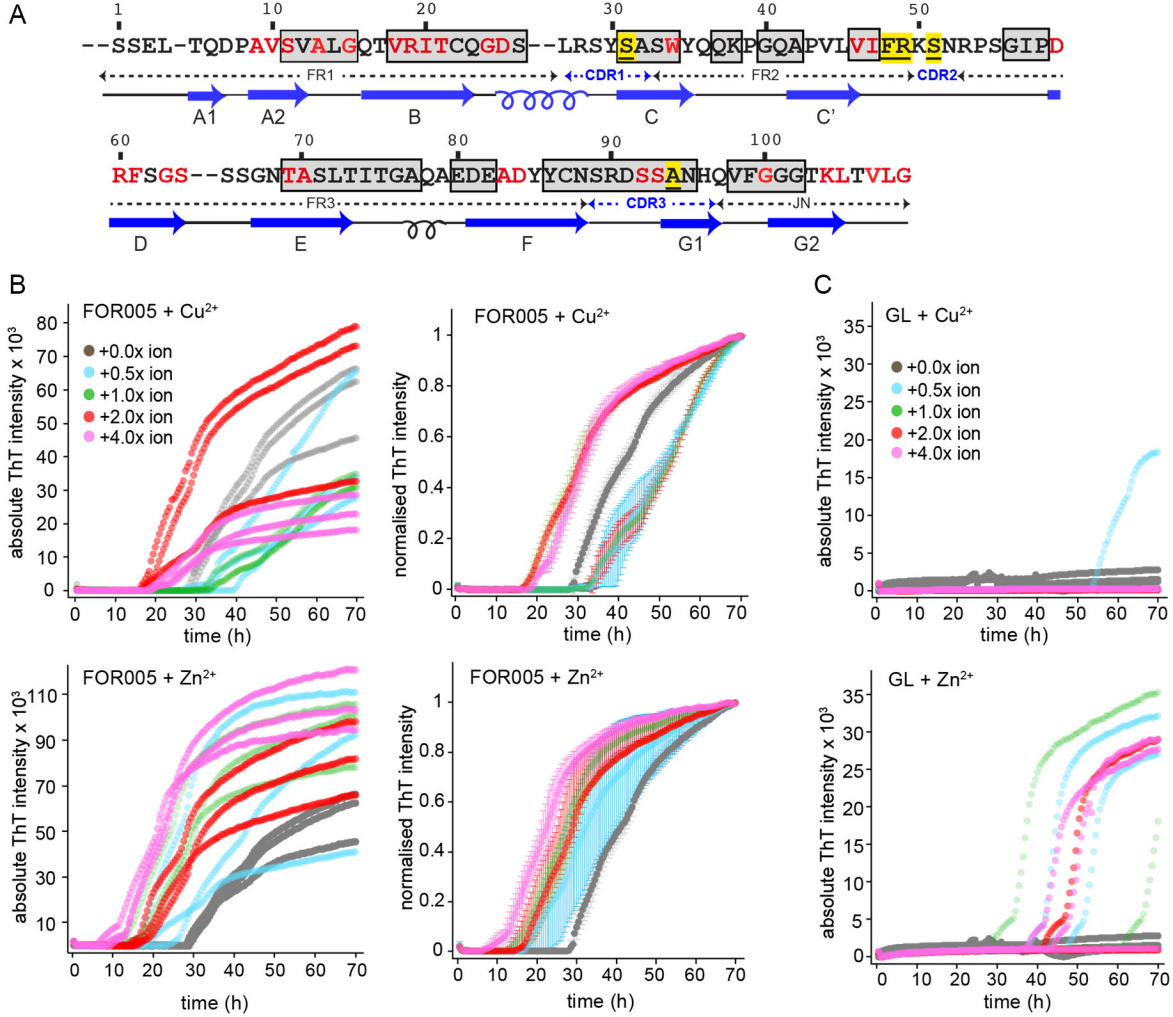
(A) FOR005 protein sequence. Residues indicated in yellow represent mutations with respect to the closest germline sequence (Y31S, Y48F, G49R, N51S, and G94A; mutations indicate transitions from GL to the patient protein). Residues color coded in red have been observed recently to be dynamic and flexible in the oligomeric state [3]. Residues highlighted with black squares could be sequentially assigned in the amyloid fibril structure [12]. ThT aggregation kinetics of (B) FOR005 and (C) GL in the presence of Cu^2+^ and Zn^2+^. The protein concentration was adjusted to 50 μM. All experiments have been recorded at 37°C. Experiments have been performed in triplicates. For the patient protein FOR005 (B), the individual experiments in absolute intensities are represented on the left, while the averages are shown on the right with normalization. For the germline protein (C), only absolute intensities are shown. In all cases, metal ions were titrated in the concentration range from 0 to 4 equivalents. The legend in the upper left panel applies to all experiments.

To characterize the effects of cationic metals on the protein aggregation kinetics, we set up a ThT aggregation assay (Figure 1). We find that high concentrations of both Cu^2+^ and Zn^2+^ decrease the lag phase which precedes amyloid fibril formation. A catalytic effect is observed for the patient protein FOR005 as well as for the GL reference. Metals thus seem to promote fibril formation. While the effect is more pronounced for the patient protein, we observe that fibril formation for the GL protein is catalyzed as well by the metal ions.

### 3.2 MD Simulation Reveals Cu^2+^ Binding Sites on the FOR005 Dimer

**FIGURE 2:**
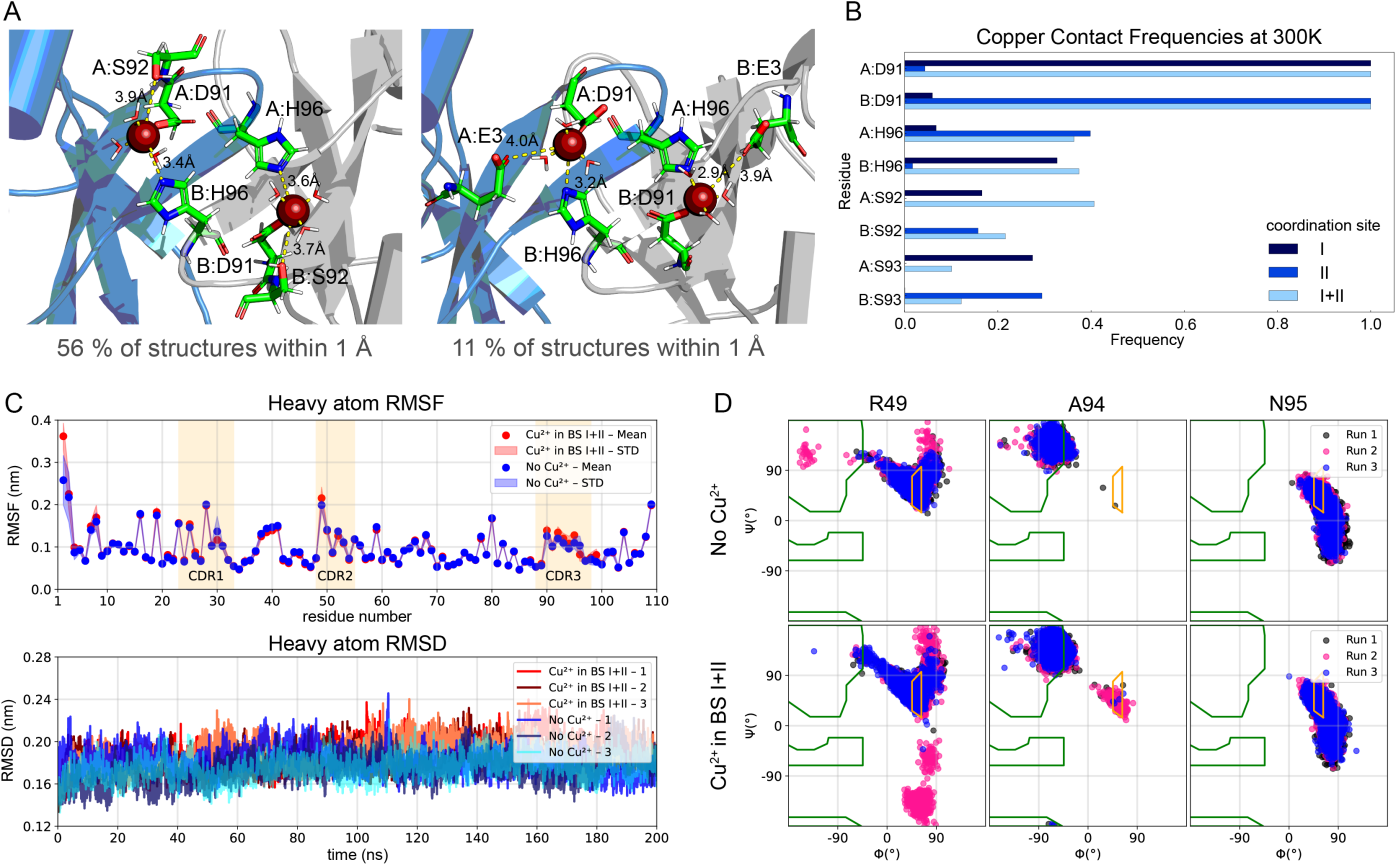
(A) Representative structures of the two most populated clusters obtained from clustering Cu^2+^‐binding geometries over 3 × 200 ns of MD simulations. (B) Residues coordinating the Cu^2+^ ions within 3.5 Å and their normalized contact frequencies from simulations initiated with Cu^2+^ in site I, site II, or both sites. A frequency of 1 corresponds to a contact over the full lengths of all trajectories. (C) RMSF of the FOR005 dimer calculated using 5L6Q as reference. Average and standard deviations are shown based on three runs for systems with Cu^2+^ in binding site I + II and without Cu^2+^. RMSD of the FOR005 dimer over the course of the MD trajectory with respect to 5L6Q. Results are shown with Cu^2+^ in binding site I + II and without Cu^2+^ for three simulations each. (D) Sampled backbone dihedral angles φ and ψ shown as Ramachandran plots for residues 49, 94, and 95 without Cu^2+^ (top) and for Cu^2+^ in binding site I + II (bottom). Favorable regions for non‐glycine residues are indicated by green contours, and a regime favorable for glycine, but less for other amino acids, is indicated in orange.

The X‐ray structure of FOR005 (PDB ID: 5L6Q) contains two zinc ions (BS I and II) that are coordinated at the interface between two protomers in the crystallographic dimer, involving residues S2, D91, S92, and H96 [38]. We performed all‐atom MD simulations to investigate the interactions of divalent Cu^2^ cations with the FOR005 dimer. In the simulations, we focus on Cu^2+^ since it has the largest effect on the NMR spectra (see below). Our simulations show that the Zn^2+^ BS identified experimentally in the dimer also accommodate Cu^2+^ (Figure 2A,B). Cu^2+^ ions that were initially placed in these sites remained stably bound throughout the simulations at both 300 and 400 K. Notably, in simulations in which Cu^2+^ ions were initially positioned outside of the BS, they diffused to and bound to the BS within 200 ns. In three independent simulations, BS I was consistently found by Cu^2+^; the second BS was simultaneously occupied in one of the three simulations. Subsequently, we analyzed three different simulation FOR005 setups: In the first setup, Cu^2+^ occupied site I in the starting structure. In the second setup, the ion was placed in site II, while two ions occupied both sites in the third simulation. In all cases, the Cu^2+^ ions were coordinated by the carboxylate oxygen of D‐91 from either monomer A or B, with a coordination distance of approximately 1.5 Å (Figure 2B). Additionally, Cu^2+^ was coordinated by three water molecules (Figure 2A), resulting in a coordination number of 4–5 rather than the typical sixfold coordination observed in bulk water [38]. In about one‐third of the simulations, the imidazole nitrogen of H‐96 of chain A/B, in addition to D‐91 from chain B/A, coordinated the Cu^2+^ ion at a distance <3.5 Å (Figure 2B). Occasional coordination by S‐92 or S‐93, as well as E‐3, was observed. The coordination geometry remained largely consistent whether one or two Cu^2+^ ions were bound.

### 3.3 Structure, Stability, and Flexibility of the FOR005 Dimer Does Not Change Significantly in the Presence of Cu^2+^

The metal BS involves residues in CDR‐3. The CDR‐3 loop is known to be in a strained conformation and the initiation point of unfolding and aggregation [3]. We therefore analyzed subsequently whether the structure and flexibility of the FOR005 dimer is affected by the binding of one or two Cu^2+^ ions. Root‐mean‐square deviation (RMSD) analysis (Figure 2C, bottom) shows that, in all cases, the dimer remains very close to the experimental structure with RMSD values consistently around 2 Å (for no, one, or two coordinated Cu^2+^ ions). Similarly, Cu^2+^ binding does not significantly alter the flexibility of the dimer. Root‐mean‐square fluctuation (RMSF) profiles as a function of residue number (Figure 2C, top) are nearly identical across all cases. We find that the dynamics is not significantly increased in the presence of Cu^2+^. Consistent with previous findings for the monomer [39], we observe that the FOR005 dimer loop adopts energetically unfavorable backbone conformations, particularly at the mutation sites G49R and N95 (Figure 2D). These conformations are unfavorable for the non‐glycine mutations and suggest local structural strain. Interestingly, the backbone dihedrals for A94 are in an unfavorable conformation for the monomer both with and without Cu^2+^ in agreement with previous MD simulations [39]. In the dimer, A94 adopts a favorable backbone conformation (Figure 2B), likely stabilized by additional interchain interactions. In the presence of Cu^2+^, the backbone transiently reverts to the unfavorable conformation in one of three trajectories. However, this brief event is unlikely to be relevant for misfolding.

To further assess the effect of Cu^2+^ binding on dimer stability, we employed the molecular mechanics Poisson–Boltzmann surface area (MM‐PBSA) method to estimate the change in binding energy between the two halves of the FOR005 dimer in the presence and absence of Cu^2+^. The calculated binding energy for the Cu^2+^‐bound dimer is approximately 5% lower than that of the apo form, indicating that Cu^2+^ binding stabilizes the dimer energetically. This stabilization arises primarily from favorable electrostatic interactions between the Cu^2+^ ions and partially negatively charged side chains within the binding pocket, such as the carboxylate oxygens of aspartate. However, these enthalpic gains are at least partially compensated by entropic contributions, including conformational changes and the entropic gain associated with the release of Cu^2+^ ions into the solvent. In summary, Cu^2+^ binding does not significantly affect the structure or flexibility of the FOR005 dimer, causing only minor local changes and a slight energetic stabilization of the dimer. The small magnitude of this stabilization suggests that Cu^2+^ does not modulate aggregation by destabilizing the native state, but rather by shifting the population of transient oligomeric intermediates.

### 3.4 NMR Experiments of Monomeric FOR005 in Solution

**FIGURE 3:**
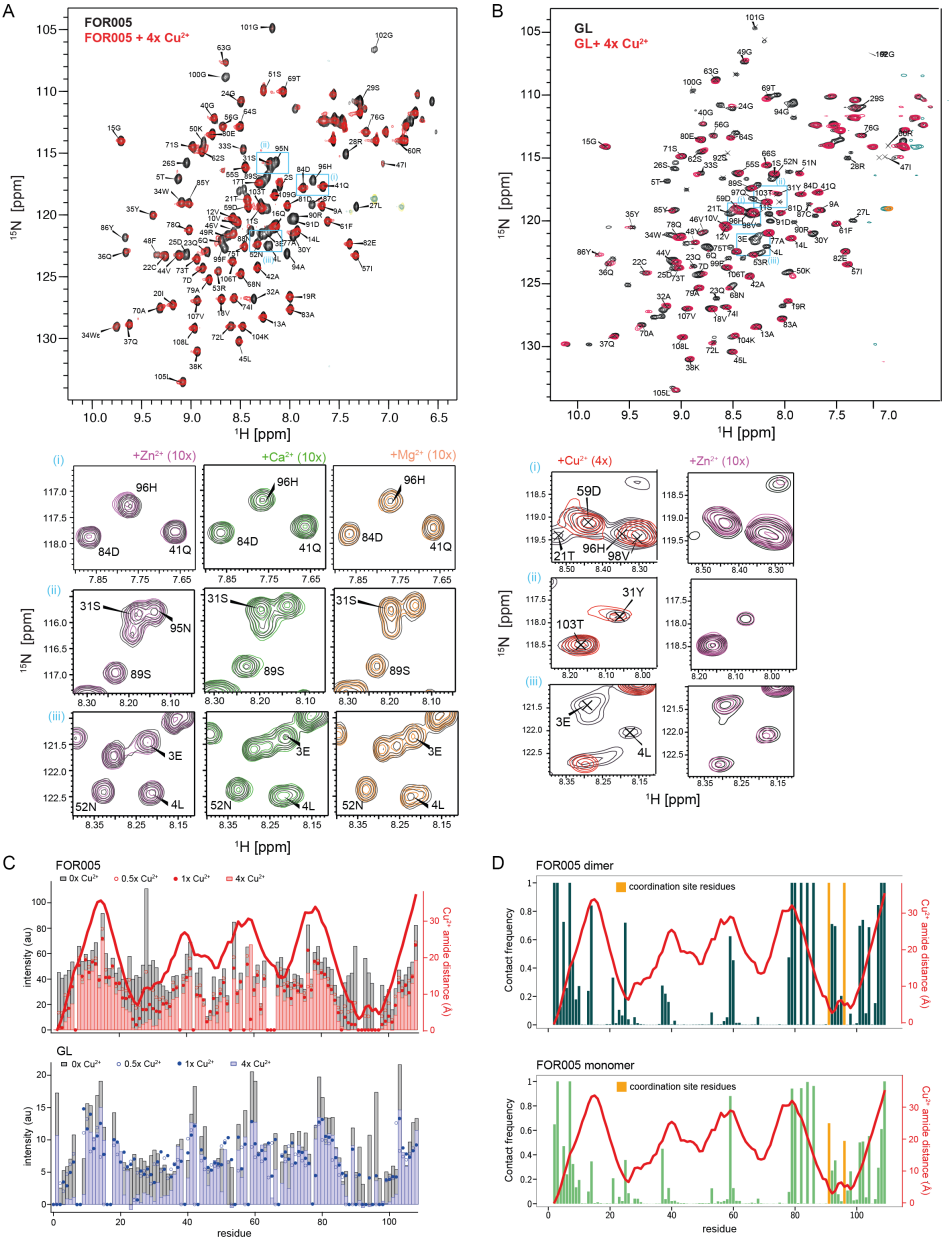
Metal binding to the monomeric antibody light chain domain FOR005. HSQC titration spectra of ^15^N labeled 50 μM sample of FOR005 (A) and GL (B) in the presence and absence of Cu^2+^, Zn^2+^, Ca^2+^, and Mg^2+^. For Cu^2+^ experiments, a 4× molar excess was employed, while the experiments involving Zn^2+^, Ca^2+^, and Mg^2+^ were carried out using a 10× molar excess. The protein concentration was adjusted to 50 μM. All experiments have been recorded at 37°C. (C) Absolute amide cross‐peak intensities for FOR005 (middle) and GL (bottom) for different Cu^2+^ concentrations. For the panel representing FOR005, the distance between amides and the crystallographic Zn^2+^ ion is represented (red solid line). (D) Residues coordinating the Cu^2+^ ions within 5 Å and their normalized contact frequencies from simulations with 150 mM CuCl_2_ of the dimer (top) and monomer (bottom). A frequency of 1 corresponds to a contact over the full lengths of all trajectories. The orange lines refer to the binding site of the dimer (residues D91 and H96).

In solution, the FOR005 V_L_ domain exists primarily as a monomer with a K_D_ for homo‐dimerization on the order of 2 mM for both the patient and GL protein [3]. We therefore asked the question how and if the metal ions interact with the FOR005 monomer. To localize the metal BS in the monomeric FOR005 protein, we titrated ions into a solution of ^15^N labeled protein and recorded ^1^H,^15^N HSQC correlation experiments (Figure 3A,B). Cu^2+^ is a paramagnetic ion that induces a line broadening and thus a reduction of the NMR resonance intensities in the vicinity of the metal BS. By contrast, Mg^2+^, Ca^2+^, and Zn^2+^ are diamagnetic ions that only induce chemical shift perturbations for amide moieties in close proximity of the metal. We observe pronounced effects on cross‐peak intensities in case the paramagnetic Cu^2+^ ion is employed for the titration, and very small chemical shift changes for Zn^2+^, while no effects are observable for Mg^2+^ and Ca^2+^. We find the largest effects for Cu^2+^, which is presumably due to the paramagnetic properties of this cation and not necessarily due to differences in binding affinity. Figure 3C shows the absolute cross‐peak intensity as a function of residue number for various molar ratios of Cu^2+^. We extracted the distance between the zinc ion and each amide moiety using the PDB ID 5L6Q and have included this information into Figure 3C. We find that the cross‐peak intensities follow the predicted distance distribution, suggesting that Cu^2+^ ions bind equally well to both FOR005 and GL in solution.

We performed MD simulations of the FOR005 monomer and dimer in 150 mM CuCl_2_ and identified all residues within 5 Å of the Cu^2+^ ions during the simulations (Figure 3D). Overall, the preferred interactions involve negatively charged oxygen‐containing residues and are similar for monomer and dimer. These preferential interactions sites correlate well with the cross‐peak intensities from the NMR experiments. We can thus rule out that Cu^2+^ induces bleaching of the NMR resonances via formation of a transient dimer. In addition, we do not find any evidence that divalent metal ions perturb the equilibrium between monomeric and dimeric FOR005 protein: The resonance frequencies of residues which are susceptible to dimerization (e.g., Q36, K38, G101, G102) are unaffected by the presence of Cu^2+^, suggesting that the equilibrium between monomer and dimer is not influenced by the metal.

### 3.5 NMR Experiments of Oligomeric FOR005 in Solution

**FIGURE 4:**
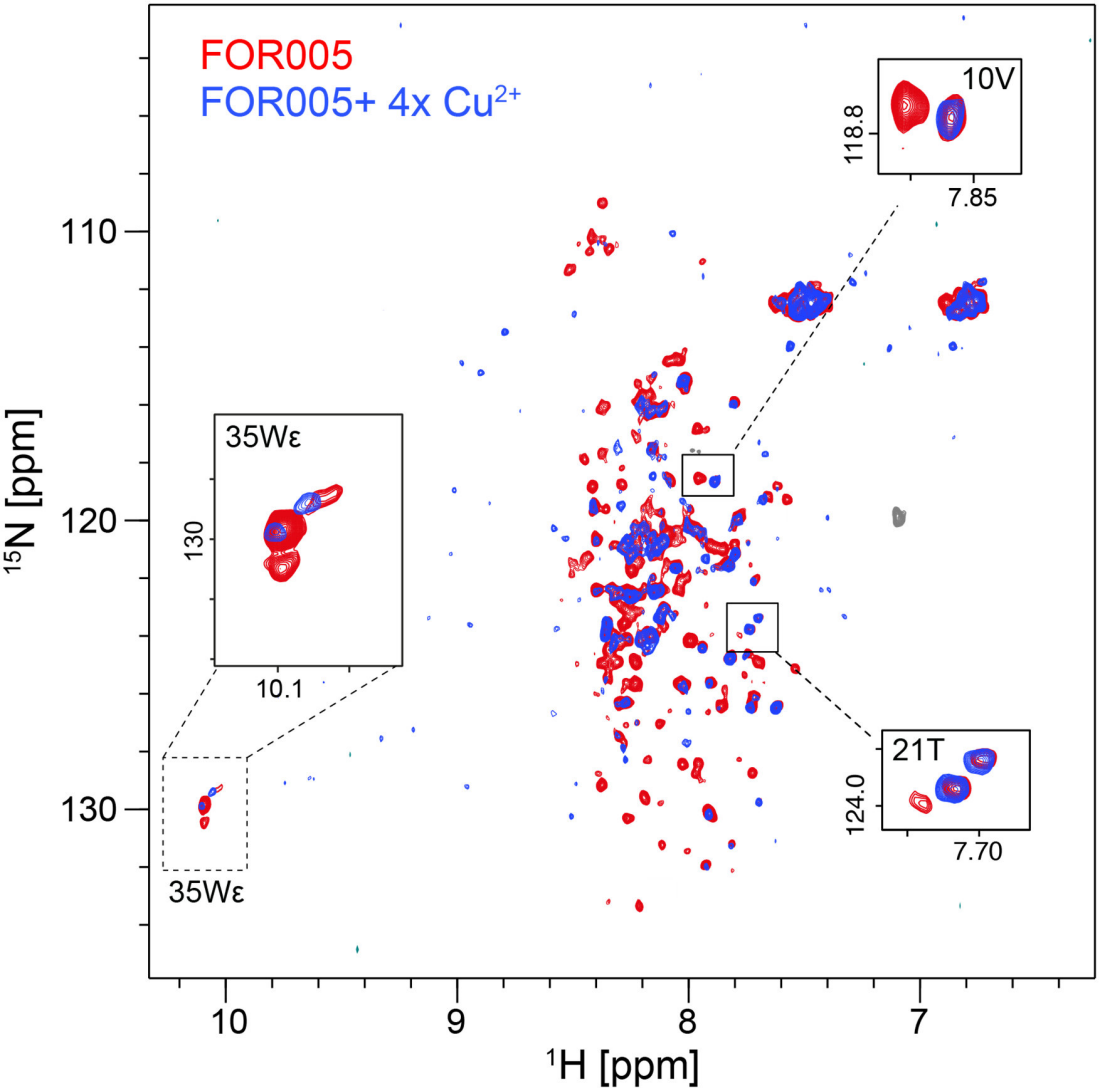
HSQC correlation spectrum of an FOR005 sample aged for 4 days in the presence and absence of a 4× molar excess of Cu^2+^. Only flexible regions of the high molecular weight oligomer are observable. The disappearance of specific peaks suggests that Cu^2+^ decreases the population of off‐pathway oligomers. Chemical shift assignments are transferred from Pradhan et al. [3].

Next, we analyzed if Cu^2+^ binds to other aggregation states of FOR005 as well. For this purpose, we prepared an aged sample of FOR005 by incubation of a freshly produced protein over a period of 4 days as described previously [3]. We divided this sample into two parts, supplemented one part with four equivalents of Cu^2+^ and recorded solution‐state HSQC experiments (Figure 4). Similar as described previously [3], we find that the sample is conformationally heterogeneous, can adopt different conformations, and forms oligomers with molecular weights on the order of 200–300 kDa. Only the flexible regions of the protein can be observed in the NMR experiments. For particular amides, four to five different cross peaks are found that possibly reflect off‐pathway intermediate state structures. For the sample prepared in the presence of Cu^2+^, we observe disappearance of many cross peaks. Missing peaks can be explained either by binding of the paramagnetic ion to specific regions of a structure or by bleaching of NMR resonances of atoms in the vicinity of the BS. Alternatively, titration of the metal might destabilize or even prevent the formation of certain off‐pathway intermediates. In fact, we find that the manifold of cross peaks that are detected for a particular amide ^1^H,^15^N correlation is reduced.

Affected residues (V10, G15, V18, T21, D25, W35ε; D59, 64S, 69T, G100) are distributed evenly across the protein sequence. This suggests that metal binding rather selects for a certain polymorph and depletes off‐pathway polymorphs. The fact that the population of off‐pathway intermediates is decreased might explain why aggregation in the presence of the metal is accelerated, since the critical concentration required to form an aggregation nucleus and convert into an amyloid fibril is reached faster. At this point, we cannot rule out that Cu^2+^ may bind to a particular polymorph and induces residue‐specific paramagnetic line broadening. Thereby, a particular aggregation intermediate state might get stabilized. The formation of amyloid fibrils is then accelerated by lowering the energetic barrier for the transition into the fibril state. The data quality, however, does not allow to differentiate between these two scenarios.

### 3.6 Interactions of FOR005 Fibrils With Cu^2+^

**FIGURE 5:**
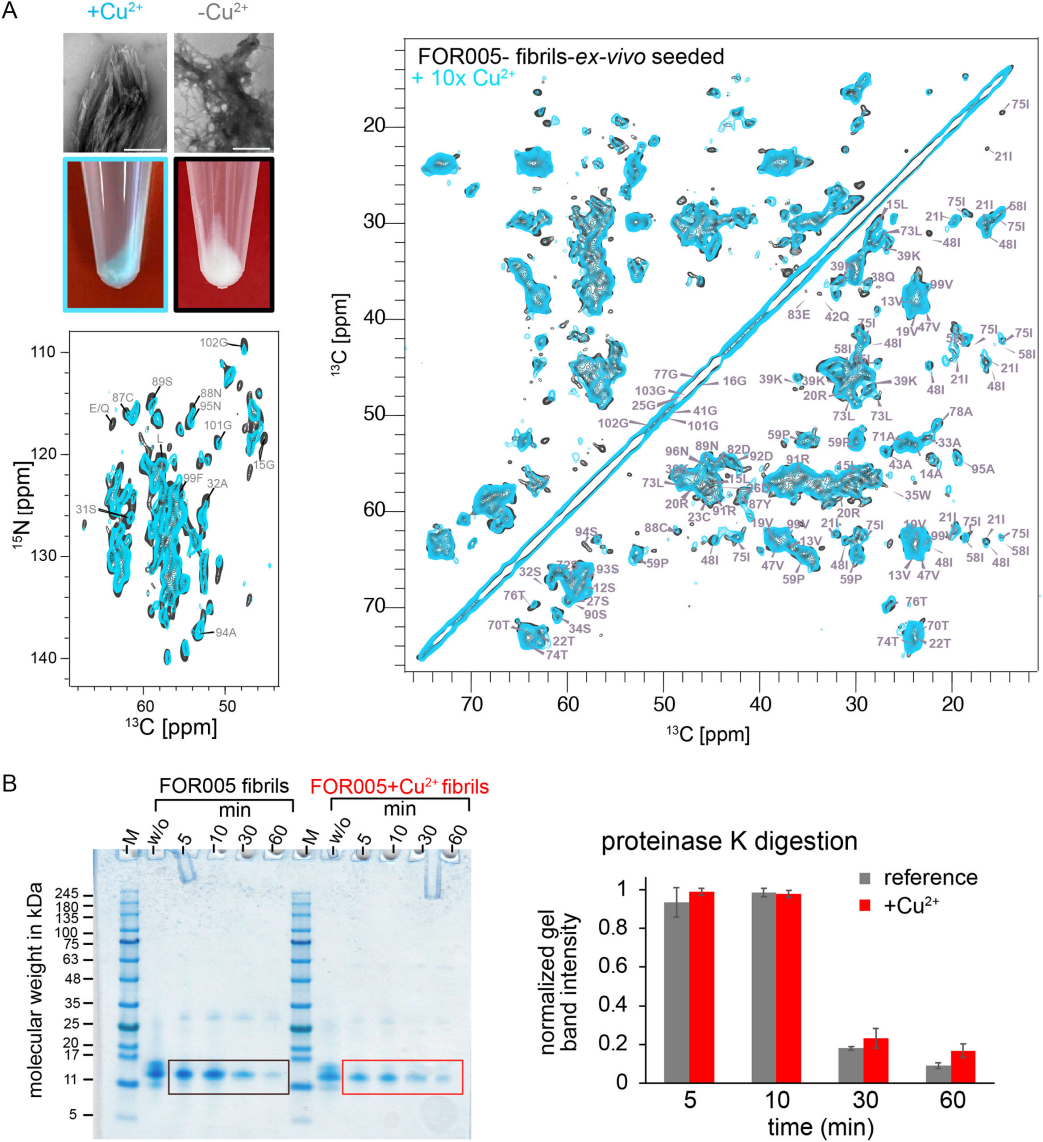
Cu^2+^ binding to preformed FOR005 fibrils. (A) FOR005 fibrils titrated with 10× Cu^2+^ appear blue, while the reference fibril sample without copper is colorless. The TEM images of the fibril preparations show small morphological differences (scale bar: 200 nm). MAS solid‐state NMR. Left: Superposition of the ^13^C,^15^N correlation spectra recorded for a fibril sample (grown using ex vivo seeds) incubated with copper (blue) and the reference sample without the metal (black). Right: Superposition of the ^13^C,^13^C correlation spectra with (blue) and without (black) Cu^2+^. Resonance assignments were obtained from fibrils grown in the absence of copper [12, 14]. (B) Proteinase K stability assay. FOR005 fibril samples were incubated for 5, 10, 30, and 60 min using proteinase K. The proteinase K assay was performed using 0.692 μM proteinase K and 8.6 μM FOR005, at a molar ratio of proteinase K to FOR005 of 1:12.5 [15]. To investigate the effect of Cu^2+^ on fibril stability, FOR005 fibrils were titrated with an equimolar concentration of Cu^2+^ ions. Left: Coomassie‐stained SDS‐PAGE gel showing the digestion of fibrils by proteinase K. The first lane (w/o) shows untreated fibrils. The assay was performed using 100 μg/mL fibril protein and 20 μg/mL proteinase K (M: molecular weight marker). Right: Normalized fibril intensity as a function of time. Intensities were quantified using the program ImageJ.

Subsequently, we analyzed how the metal ions interact with amyloid fibrils. For this purpose, we titrated Cu^2+^ in a 10× molar excess to a preformed FOR005 fibril sample and recorded MAS solid‐state NMR experiments. We find that the fibril pellet adopts a blue color (Figure 5A), suggesting that the metal ions bind to the amyloid fibrils. On the other hand, solid‐state NMR spectra show only marginal differences in the presence and absence of the metal. In particular, no resonances are disappearing which would be expected in the presence of the paramagnetic ion.

Potentially, metal ions could bind to residues which were previously not detectable (residues 1–10; CDR2: 48–54, 59−68; 103–109) [12]. In order to find out whether copper stabilizes the amyloid fibril fold, we carried out a proteinase K digestion assay. FOR005 fibrils were incubated for 5, 10, 30, and 60 min using proteinase K, as described in Schönfelder et al. [15]. As expected, the gel bands yield decreasing intensities for prolonged incubation with the protease. We find that the gel band intensities disappear more slowly in the presence of one equivalent Cu^2+^ ions suggesting that the metal in fact increases the fibril stability against proteolytic digestion.

**FIGURE 6:**
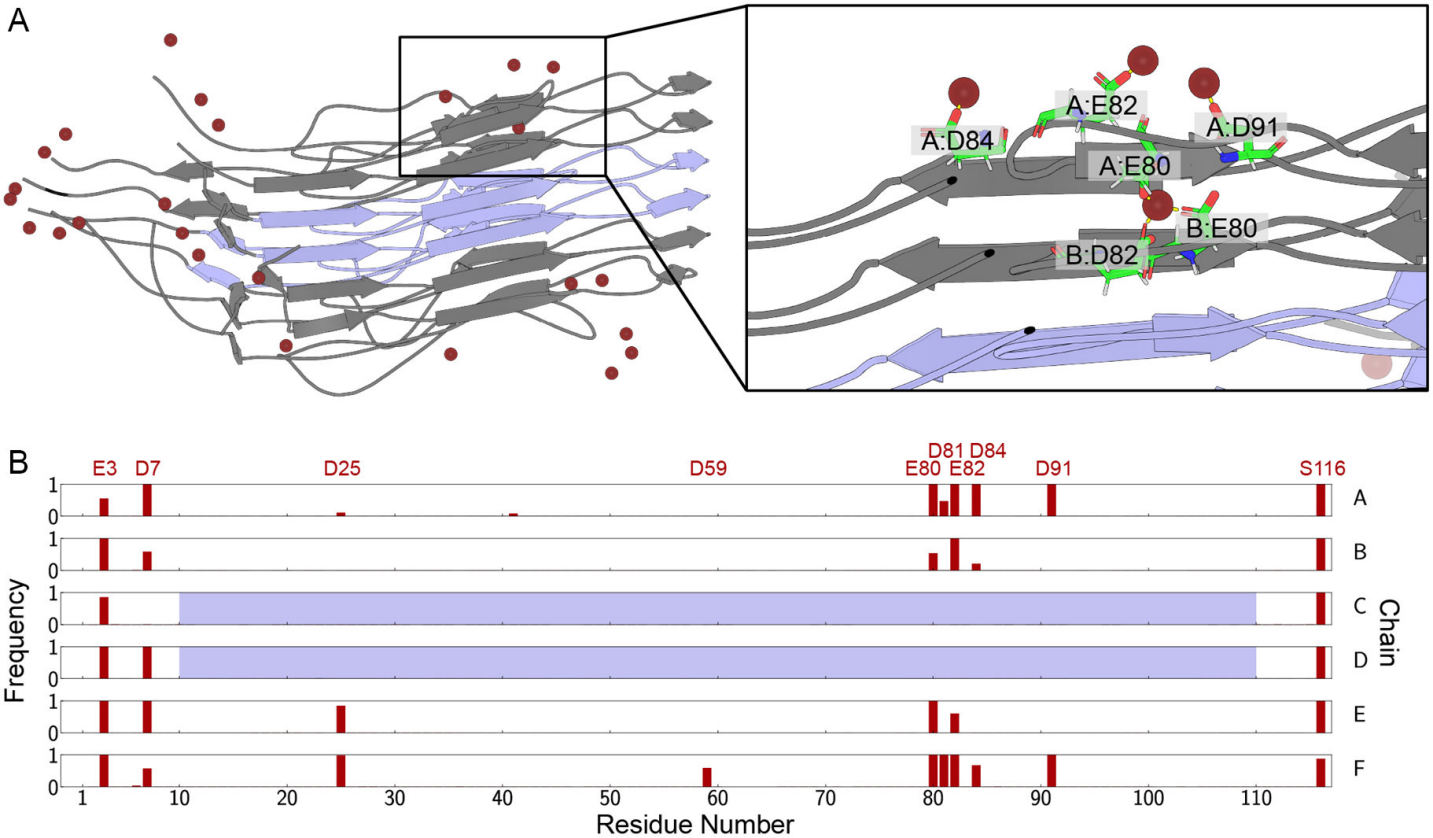
(A) Representative snapshots after 200 ns of a MD simulation of the FOR005 fibril in 150 mM CuCl_2_. The fibril core region is colored light blue in all representations. Only Cu^2+^ ions in contact with the fibril are shown as dark red spheres. The inlet shows representative Cu^2+^ coordination in atomistic detail. (B) Cu^2+^ coordinating residues within 0.35 nm and their contact frequency.

To rationalize the NMR results, we carried out MD simulations involving the FOR005 fibril in the presence of Cu^2+^. We could show previously [12] that the topology of the amyloid in the solid‐state NMR preparation is the same as in the cryo‐EM study [40]. The simulations reveal a Cu^2+^ binding pattern along the fibril surface (Figure 6). Similar to the monomer and dimer, the primary interaction partners are aspartate and glutamic acid side‐chain oxygens. The Cu^2+^ ions preferentially bind in three different locations, involving the N‐ and C‐terminal residues, as well as the loop region of the protein chains at the fibril tips. For the former two, the Cu^2+^ ions are coordinated by only one oxygen or nitrogen atom. In the loop region, up to two contacts can form (Figure 6), bridging interactions between adjacent peptide chains. Importantly, we do not find Cu^2+^ binding to the inner core of the fibril or to the β‐sheet surface further away from the fibril tips. This is largely consistent with the NMR data, which do not yield a specific Cu^2+^ BS. Cu^2+^ affects all resonances in a similar way, and no dramatic changes in peak intensity are observed for specific residues. Furthermore, terminal residues are not observable in the solid‐state NMR experiments. The simulations indicate that Cu^2+^ does not bind to a specific site but interacts nonspecifically with the termini of the peptide chains which can bind many Cu^2+^ ions. As such, the fibrils act as a reservoir accommodating many metal ions.

## 4 Conclusions

In this study, we examined how the divalent metal ions Cu^2+^ and Zn^2+^ influence the aggregation behavior of the amyloidogenic λ‐III AL variable domain FOR005 and its GL counterpart. Cu^2+^ yields the strongest effects in NMR experiments due to the paramagnetic nature of the ion. For both metals, we observe a shortening of the lag phase preceding fibril formation in ThT assays, indicating a metal‐induced enhancement of the aggregation kinetics at high concentrations.

MD simulations identified two Cu^2+^ BS at the dimer interface, coordinated primarily by D‐91 and H‐96 residues. Despite stable coordination, Cu^2+^ binding caused only minor local structural changes and a slight energetic stabilization of the dimer, showing that the native fold remains intact. MD shows that Cu^2+^ interactions preferably involve negatively charged oxygen‐containing residues and are similar for monomer and dimer. For both monomeric and dimeric FOR005, dynamics in particular in the CDR‐3 loop region is unaffected by metal binding and cannot explain the changes in the rate of the aggregation kinetics. The MD results are consistent with solution‐state NMR experiments data and confirm the preferred interaction sites of the metal ions.

By contrast, Cu^2+^ binding seems to reduce the population of off‐pathway oligomeric intermediates, suggesting a shift toward productive aggregation. Cu^2+^ ions are particularly well‐suited for modulating intermediate states, as they exhibit both high binding affinity and fast exchange kinetics. The affinity of metal ions for carboxylate oxygens increases in the order Ca^2+^ < Mg^2+^ ≤ Zn^2+^ < Cu^2+^ [41], whereas exchange rates follow Mg^2+^ < Zn^2+^ < Ca^2+^ < Cu^2+^ [42]. Consequently, Cu^2+^ can transiently stabilize both on‐ and off‐pathway intermediates, leading to the observed accelerated aggregation kinetics.

Our experiments indicate super‐stoichiometric binding to amyloid fibrils and a decreased susceptibility to proteinase K digestion in presence of Cu^2+^. The N‐ and C‐termini of the protein are identified as BS for metals by MD. No defined BS could be identified for the fibril state in solid‐state NMR experiments. The super‐stoichiometric binding mode of copper implies that aggregates might act as a binding reservoir in the cell for this metal.

Independent of their binding affinities to native LC protein, other ions such as Zn^2+^, Mg^2+^, or Ca^2+^ might influence the protein aggregation kinetics and associate with misfolding intermediates or amyloid fibrils. The high cellular concentration of these ions and the abundance of LC protein in the plasma might hint toward a role of protein aggregate–associated metal ions in disease. A decrease of the cytosolic free pool of ions would perturb cellular ion homeostasis in cardiomyocytes which in turn would result in dysregulated metabolic function. Future studies will have to show whether these metal ions affect the aggregation kinetics and bind to oligomeric or aggregated LC protein.

Together, our findings support a model in which Cu^2+^ accelerates aggregation by differentially stabilizing transient intermediates, either by reducing the population of off‐pathway species or by shortening the lifetimes of on‐pathway states, ultimately promoting fibril formation and stability.

## Funding

This work was supported by Deutsche Forschungsgemeinschaft (Re1435/26‐1, 529538247, and 499211671) and Erlangen National High Performance Computing Center (b119ee and b253ee).

## Conflicts of Interest

The authors declare no conflicts of interest.

## Data Availability

The data that support the findings of this study are available from the corresponding author upon reasonable request.
